# Regional Disparities in Factors Associated With Subjective Health Among Older Adults in Aging and Super-Aged Areas of Korea: Nationwide Cross-Sectional Study

**DOI:** 10.2196/80189

**Published:** 2026-03-02

**Authors:** Gi-Moon Nam, Ye-Rin Lee, Ji-Hye Mun, Su-Hyeun Cho, Hee-Sook Lim

**Affiliations:** 1 AgeTech-Service Convergence Major, Department of Gerontology, Graduate School of East-West Medical Science, Kyung Hee University, Deogyeong-daero 1732, Giheung-gu, Yongin-si, 17104, Republic of Korea, 82 031-201-2936; 2 Dietary and Nutritional Safety Policy Division, Food and Consumer Safety Bureau, Ministry of Food and Drug Safety, Cheongju, Republic of Korea

**Keywords:** subjective health status, health literacy, older adult, chronic disease, health disparities

## Abstract

**Background:**

As South Korea transitions into a super-aged society, understanding regional disparities in subjective health among older adults is critical to addressing health inequalities and supporting healthy aging.

**Objective:**

This study aimed to compare determinants of subjective health between aging and super-aged areas in South Korea and identify region-specific characteristics contributing to disparities among older adults.

**Methods:**

A cross-sectional analysis was conducted using data from the Korea Community Health Survey (2020‐2023), a nationwide population-based survey at the city, county, and district levels. Adults aged 65 years and older (n=179,571) were categorized into aging (n=19,759) or super-aged (n=159,782) areas based on regional aging rates. Propensity score matching was applied to adjust for demographic differences, yielding 18,574 matched participants in each group. Subjective health was assessed using a 5-point Likert scale. Ordinal logistic regression was used to examine associations between subjective health and various exposures, including demographic characteristics, health behaviors, physical and mental health status, and health literacy indicators such as nutrition label recognition and reading.

**Results:**

Older adults in super-aged areas reported poorer subjective health than those in aging regions. Physical activity and mental health were consistently associated with better subjective health in both regions, region-specific patterns were observed. In aging regions, nutrition label recognition was significantly associated with better subjective health, whereas in super-aged areas, nutrition label reading showed a stronger association. The negative impact of hypertension and diabetes on subjective health was more pronounced in super-aged areas.

**Conclusions:**

Although key determinants of subjective health were similar across regions, regional differences underscore the importance of tailored public health strategies. Interventions that strengthen health literacy and provide nutrition education focused on disease-related nutrients may help mitigate disparities and enhance subjective health among older adults in aging and super-aged areas.

## Introduction

South Korea’s older population has increased by approximately 4.4% per year in recent years, reflecting one of the fastest aging trends among Organization for Economic Co-operation and Development (OECD) countries [1]. In December 2024, the proportion of individuals aged 65 and older exceeded 20%, and South Korea was classified as a super-aged society [2]. This transition occurred just 7 years after entering an aged society, a steeper trajectory than Japan’s 12-year process [3], and highlights the need for rapid societal adaptation.

Among older adults, the focus has shifted from simply extending life expectancy to increasing healthy life expectancy, meaning living longer in good health [4]. Reducing the gap between these 2 indicators by minimizing illness periods has become a key public health issue. In 2019, South Korea’s life expectancy was 83.3 years, and healthy life expectancy was 73.1 years, indicating a 10.2-year gap, exceeding the OECD average of 9.6 years [5]. A 2-year difference between the top 20% and bottom 20% of regions within the country further underscores the need to reduce regional disparities [6].

The health status of older adults, a key determinant of healthy life expectancy, is influenced by a range of factors, including socioeconomic and behavioral factors [7]. Objective health is typically defined as measurable clinical indicators, such as clinical diagnoses or physiological functioning, whereas subjective health refers to individuals’ self-assessment of their overall health status. Because objective health is difficult to measure directly, subjective health is often used as a proxy indicator [8]. South Korea scored 52.4 in subjective health, ranking 28th among 38 OECD countries and below the OECD average of 67.6 [9]. Similar to objective health, subjective health is associated with sociodemographic characteristics, physical and mental health, and health behaviors. Individuals who live with others, participate in economic activity, earn higher incomes, and have higher education levels tend to report better subjective health [10-13]. Smoking, low physical activity, depression, and chronic disease diagnoses have also been associated with poorer subjective health [14-16].

Environmental factors associated with older adults’ health include regional attributes such as access to medical services, economic conditions, and housing, as well as social environments including neighborhood relationships and health-supportive community settings [17-19]. These factors may be related to or shape health behaviors within everyday living contexts. However, most studies rely on broad regional classifications, such as metropolitan versus nonmetropolitan or urban versus rural, which limit the analysis of local variation.

Using data from the Korea Community Health Survey (KCHS), which produces community-level health statistics, this study aimed to analyze factors influencing the subjective health of older adults by comparing aging and super-aged areas and considering relevant environmental determinants.

## Methods

### Study Design and Setting

This study employed a cross-sectional design using nationally representative data from the KCHS, conducted annually by the Korea Disease Control and Prevention Agency (KDCA). The KCHS is the largest nationwide health survey in Korea, including approximately 230,000 participants each year and representing about 0.5% of the country’s adult population. It provides regionally disaggregated data that enable community-level health analyses. The sampling frame was established by linking resident registration data from the Ministry of the Interior and Safety with housing data from the Ministry of Land, Infrastructure and Transport to construct a complete list of households.

The survey used a multistage, stratified probability sampling method. In the first stage, primary sampling units (PSUs) were selected based on administrative districts (tong/ban/ri) with probabilities proportional to the number of households by housing type. In the second stage, households were systematically sampled within each PSU. The overall sample size was predetermined by the national survey design, and approximately 10 households per PSU in urban areas and 8 households per PSU in rural areas were selected to account for regional variations in health indicators and sampling adequacy.

Trained interviewers visited selected households and collected data through computer-assisted personal interviewing using tablet-based electronic questionnaires between August and October each year.

Since 2010, the KCHS has been organized into 4-year survey cycles, with items categorized according to importance, applicability, and sampling size. Accordingly, certain items are assessed every 1, 2, or 4 years. The total number of questionnaire items ranged from approximately 140 to 160 per year (142 in 2020, 163 in 2021, 138 in 2022, and 145 in 2023), with minor revisions made annually to reflect changes in public health priorities and regional conditions.

### Subjects and Data Collection

**Figure 1.**
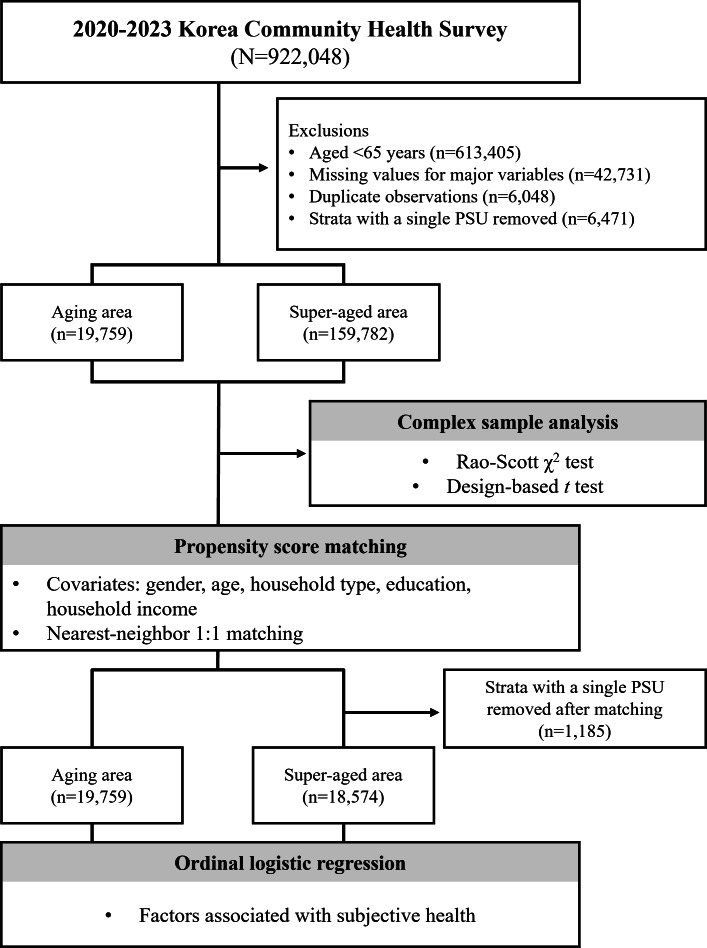
Conceptual framework of the study. Under “Exclusions,” major variables included demographic characteristics, health behavior characteristics, and health literacy variables (see Tables 1 and 2). Aging area has an aging rate ≤14%, while super-aged area has an aging rate ≥20%. PSU: primary sampling unit.

This study used merged KCHS data from 2020 to 2023, including 922,048 participants, to examine factors influencing subjective health by regional aging levels. Exclusions included 613,405 individuals aged 65 years or younger, 42,731 with missing or outlier values, 6048 with duplicated identifiers, and 6471 from strata with only 1 PSU, resulting in a final sample of 179,571. Regions were classified by linking each respondent’s location to aging rate data from Statistics Korea at the Si-Gun-Gu (city-county-district) level. Areas with aging rates of 14% or lower were defined as “Aging area” (n=19,759), and those with aging rates of 20% or higher were defined as “Super-aged area” (n=159,782), based on commonly used demographic thresholds that have been applied in national-level classifications as well as regional analyses in Korea [20]. The study design and analytical procedures are summarized in Figure 1.

### Research Variables

Subjective health was assessed using a 5-point Likert scale ranging from “Very bad” to “Very good” (1=Very bad, 2=Bad, 3=Fair, 4=Good, 5=Very good). Respondents were asked the question, “In general, how would you rate your health?.” To identify the factors influencing subjective health, items from the KCHS related to demographic characteristics, health behavior characteristics, health status characteristics, and health literacy were used.

### Demographic Characteristics

Demographic variables included gender, age (years), household type, marital status, education, economic activity, and household income (monthly). Specifically, household type was categorized into “living alone,” “with a spouse or other (single-generation household),” “two generations (couple with unmarried children, single parent with unmarried children, or other 2-generation households),” and “three generations.” Marital status was divided into “yes, cohabitation,” “separated or divorced,” “bereavement,” and “no.” Education was classified into “below elementary (including no formal education or traditional education),” “elementary,” “middle school,” “high school,” and “college or higher (2-y or 3-y college, 4-y university, graduate school or higher).” Monthly household income was reported as a continuous variable.

### Health Behavior Characteristics

Health behavioral characteristics included smoking (yes), drinking frequency, active physical activity (weekly, min), moderate physical activity (weekly, min), walking (weekly, min), subjective body shape, and weight control experience. Smoking was categorized into “yes” and “nonsmoker (smoked fewer than 5 packs or never smoked).” Drinking frequency was classified into “almost never (no drinking experience or none within the past year),” “less than once a month,” “1‐4 times a month (once or 2‐4 times per month),” and “more than 2 times a week (2‐3 times or more than 4 times per week).” Physical activity (active, moderate, and walking) was calculated as a continuous variable by multiplying the average daily minutes spent on each activity by the number of days it was performed per week, resulting in total weekly minutes.

### Health Status Characteristics

Health-related characteristics included subjective stress, depression, hypertension, diabetes, subjective health, chewing discomfort, and subjective oral health. Specifically, chewing discomfort was classified into “uncomfortable (very uncomfortable, uncomfortable),” “neutral,” and “comfortable (slightly uncomfortable, not uncomfortable at all).”

### Health Literacy

Health literacy variables included nutrition label recognition, nutrition label reading, nutrition label utilization, unmet health care needs, and written health literacy. Nutrition label items were structured hierarchically to represent sequential levels of label literacy: recognition (awareness of labels on food packaging), reading (understanding the information displayed), and utilization (using label information in dietary decisions). Unmet health care needs were categorized into 2 groups: “yes” (excluding cases indicating no need for care) and “no.”

### Statistical Analysis

Statistical analyses were performed using RStudio (version 4.3.3; Posit, PBC) and IBM SPSS Statistics (version 25.0; IBM Corp.). A complex sample survey analysis method was applied considering the individual weights, strata, and cluster variables provided by the KCHS. These weights were calculated as the inverse of the selection probability and adjusted for response rates and population structure to represent the Korean adult population. Categorical variables were summarized as frequencies and percentages, and group differences were tested using the Rao-Scott chi-square test. Continuous variables were presented as mean (SD) and compared using design-based *t* tests. Statistical significance was defined as *P*<.05.

Propensity scores were calculated using 5 variables: gender, age, household type, education, and household income (monthly). Based on these propensity scores, data from aging and super-aged areas were matched using a 1:1 nearest-neighbor matching method. The covariate balance between the matched groups was confirmed by comparing standardized mean differences before and after matching. Postmatching weights were calculated using poststratification adjustment based on original KCHS sampling weights and applied in subsequent analyses [21].

Ordinal logistic regression analysis was conducted using subjective health as the dependent variable. The backward elimination method of stepwise selection was applied, starting with a full model containing all predictors and sequentially removing variables with the highest nonsignificant *P* values. The threshold for variable removal was set at *P*>.10. After each removal, the model was refitted and the *P* values were recalculated. This process was repeated until all the remaining variables reached statistical significance.

To identify significant regional differences in determinants of subjective health, subgroup analyses were performed separately for aging and super-aged areas using the same model structure. Odds ratios (OR) and 95% CI were estimated for each group. Subsequently, a *z* test was performed to compare the OR and SDs between the regions. The significance level used to identify trends in regional differences was set at 90% (*P*<.10).

### Ethical Considerations

This study was conducted using secondary data from KCHS provided by the KDCA. In accordance with Article 2(2) of the Enforcement Rule of the Bioethics and Safety Act [22], the study was exempt from institutional review board review because it did not involve direct human participation. The original data were collected under the Regional Public Health Act and its Enforcement Decree to support evidence-based community health programs. All participants in the KCHS provided written informed consent that included information about the purpose of data collection, the types of personal information gathered, and the period of data retention and use.

The dataset supplied by the KDCA was deidentified in accordance with the Personal Information Protection Act, using pseudonymization procedures in which identifiers such as sample point numbers and household numbers were replaced with numerical codes to prevent personal identification. Participants in the original survey received small incentives, including a gift voucher worth approximately KRW 10,000 (US $7) and additional daily necessities, provided by local community health centers. No identifiable images or materials that could reveal participants’ identities were used in this study.

## Results

### Propensity Score Matching

**Figure 2.**
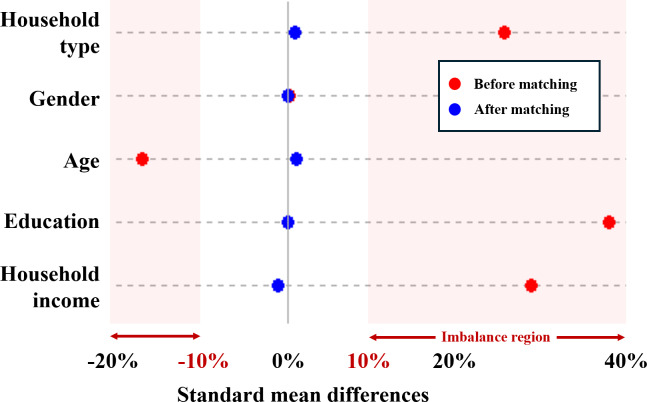
Covariate changes before and after propensity score matching. An absolute standardized mean difference (|SMD|>0.10) indicates covariate imbalance (highlighted in red). SMD was used to assess covariate balance after matching.

Of the 159,782 respondents in super-aged areas, 19,759 were matched 1:1 to those in aging areas using nearest-neighbor matching. After excluding strata with a single PSU, the final matched dataset included 18,574 individuals. All covariates achieved standardized mean differences below 0.10 [23], including gender (<0.001), age (0.01), household type (0.009), education (<0.001), and household income (−0.01), confirming an adequate covariate balance between groups (Figure 2).

### Comparison of Demographic Characteristics After Matching

After matching, demographic characteristics were compared between aging and super-aged areas, including both the variables used for matching and additional variables not used in the matching process, such as age groups, marital status, and economic activity (Table 1). Comparisons of the same variables between aging and super-aged areas before propensity score matching are provided in Table S1 in Multimedia Appendix 1. Marital status and participation in economic activity differed significantly between regions (both *P*<.001).

**Table 1.** Demographic characteristics of older adults (≥65 y) in aging and super-aged areas of Korea after propensity score matching.

Variable	Matched data^a^		
	Aging area^b^ (n=19,759)	Super-aged area (n=18,574)	*P* value^c^ (SMD)^d^
Gender, n (%)			.11 (<.001)^d^
Male	2,216,305 (45.7)^3^	1,169,928 (44.5)	
Female	2,654,450 (54.3)	1,457,693 (55.5)	
Age (y), mean (SD)	73.3 (6.6)	73.2 (6.5)	.29 (.01)^d^
Age (y), n (%)			.36
65‐74	3,016,953 (61.7)	1,641,793 (62.5)	
Over 75	1,871,773 (38.3)	985,828 (37.5)	
Household type, n (%)			<.001 (.009)^d^
Living alone	956,634 (19.6)	509,564 (19.4)	
With a spouse or other	2,357,156 (48.2)	1,368,420 (52.1)	
2 generations	1,110,306 (22.7)	538,923 (20.5)	
3 generations	464,630 (9.5)	210,713 (8.0)	
Marital status, n (%)			<.001
Yes, cohabitation	3,158,471 (64.6)	1,763,107 (67.1)	
Separation or divorced	390,972 (8.0)	158,176 (6.0)	
Bereavement	1,303,745 (26.7)	689,221 (26.2)	
No	35,538 (0.7)	17,117 (0.7)	
Education, n (%)			.006 (<.001)^d^
Under elementary	379,104 (7.8)	223,548 (8.5)	
Elementary	1,415,418 (29.0)	821,941 (31.3)	
Middle school	1,005,594 (20.6)	513,614 (19.5)	
High school	1,300,113 (26.6)	679,110 (25.8)	
College or over	788,496 (16.1)	389,409 (14.8)	
Economic activity, yes, n (%)	1,316,003 (26.9)	987,473 (37.6)	<.001
Household income, 1 million KRW^e^, mean (SD)	262.7 (247.3)	242.8 (236.0)	<.001 (–.01)^d^

a Propensity score matching was performed using gender, age, household type, education, and household income.

b Aging areas were defined as regions with aging rates ≤14%, and super-aged areas as those with ≥20%.

c Rao-Scott chi-square tests for categorical variables, independent sample *t* tests for continuous variables.

d Standardized mean differences (SMD) for variables used in propensity score matching.

e 1 million KRW=US $700.

### Comparison of Variables Between 2 Groups

Table 2 summarizes regional differences in health behaviors, health status, and health literacy. Among health behaviors, drinking frequency differed significantly between regions, with respondents in the super-aged areas showing more polarized drinking patterns and slightly higher rates of frequent drinking (*P*=.02). Smoking prevalence was similar across regions and was not significantly different. Regarding chronic conditions, the prevalence of depression was significantly lower in the super-aged areas (*P*=.003), whereas hypertension and diabetes did not differ notably between regions. Subjective health status and subjective oral health were poorer among respondents in the super-aged areas (*P*<.001 and *P*=.004, respectively). For health literacy, recognition of nutrition labels was higher in the super-aged areas (*P*=.002), but reading and utilization rates were lower (*P*<.001 and *P*=.03, respectively), suggesting difficulties in applying nutrition information in daily practice.

**Table 2.** Health-related characteristics of aging areas and super-aged areas after matching.

Variable	Matched data^a^		
	Aging area^b^ (n=19,759)	Super-aged area (n=18,574)	*P* value^c^
Smoking, n (%)			.97
Yes	1,763,544 (36.1)	947,086 (36.0)	
Nonsmoker	3,125,182 (63.9)	1,680,535 (64.0)	
Drinking, n (%)			.02
Almost never	2,986,748 (61.1)	1,621,490 (61.7)	
Less than once a month	581,809 (11.9)	283,298 (10.8)	
1‐4 times a month	762,930 (15.6)	391,855 (14.9)	
More than 2 times a week	557,239 (11.4)	330,977 (12.6)	
Active physical activity (weekly, min), mean (SD)	38.8 (182.1)	47.0 (216.6)	.02
Moderate physical activity (weekly, min), mean (SD)	84.0 (263.1)	133.3 (385.1)	<.001
Walking (weekly, min), mean (SD)	273.7 (334.3)	250.9 (347.0)	<.001
Subjective body shape, n (%)			<.001
Very thin	128,784 (4.2)	104,497 (4.8)	
Slightly thin	462,204 (15.1)	376,489 (17.3)	
Average	1,493,374 (48.6)	1,074,713 (49.4)	
Slightly obese	885,780 (28.9)	558,236 (25.7)	
Very obese	99,544 (3.2)	59,644 (2.7)	
Weight control experience, n (%)			<.001
Tried to lose weight	1,349,787 (27.6)	607,446 (23.1)	
Tried to maintain	979,810 (20.0)	463,944 (17.7)	
Tried to gain weight	274,146 (5.6)	179,628 (6.8)	
Never tried	2,284,983 (46.7)	1,376,603 (52.4)	
Subjective stress, n (%)			.84
Extremely stressed	91,665 (1.9)	46,217 (1.8)	
Much stressed	642,962 (13.2)	340,560 (13.0)	
Little stressed	2,353,430 (48.1)	1,257,394 (47.9)	
Stressless	1,800,669 (36.8)	983,450 (37.4)	
Depression, yes, n (%)	420,954 (8.6)	191,963 (7.3)	.003
Hypertension, yes, n (%)	2,548,124 (52.1)	1,377,820 (52.4)	.70
Diabetes, yes, n (%)	1,171,928 (24.0)	648,287 (24.7)	.33
Chewing discomfort, n (%)			.11
Uncomfortable	1,478,303 (30.2)	798,880 (30.4)	
Neutral	935,398 (19.1)	467,012 (17.8)	
Comfortable	2,475,025 (50.6)	1,361,728 (51.8)	
Subjective oral health, n (%)			.004
Very good	101,933 (2.1)	58,141 (2.2)	
Good	866,235 (17.7)	456,730 (17.4)	
Fair	1,885,743 (38.6)	956,906 (36.4)	
Bad	1,580,120 (32.3)	925,295 (35.2)	
Very bad	454,695 (9.3)	230,549 (8.8)	
Subjective health, n (%)			<.001
Very good	209,405 (4.3)	84,084 (3.2)	
Good	1,329,045 (27.2)	674,556 (25.7)	
Fair	2,057,748 (42.1)	1,078,442 (41.0)	
Bad	1,025,139 (21.0)	622,846 (23.7)	
Very bad	267,389 (5.5)	167,692 (6.4)	
Nutrition label recognition, n (%)	1,891,633 (38.7)	1,086,721 (41.4)	.002
Nutrition label reading, n (%)	759,477 (15.5)	375,564 (14.3)	<.001
Nutrition label utilization, n (%)	612,869 (12.5)	292,871 (11.1)	.03
Unmet health care needs, n (%)			.26
Yes (unmet)	189,944 (4.1)	110,665 (4.5)	
No (met)	4,489,698 (95.9)	2,374,057 (95.5)	
Written health literacy, n (%)			<.001
Very easy	106,457 (8.0)	62,850 (10.5)	
Somewhat easy	572,592 (43.1)	223,136 (37.1)	
Somewhat difficult	289,349 (21.8)	125,325 (20.8)	
Very difficult	82,198 (6.2)	31,535 (5.2)	
No interest	279,095 (21.0)	158,437 (26.3)	

a Propensity score matching was performed using gender, age, household type, education, and household income.

b Aging areas were defined as regions with aging rates ≤14%, and super-aged areas as those with ≥20%.

c Rao-Scott chi-square tests for categorical variable, independent sample *t* tests for continuous variable.

### Results of Ordinal Logistic Regression

Ordinal logistic regression was first performed on the overall matched data to identify factors associated with subjective health. The results, presented in Table S2 in Multimedia Appendix 2, showed consistent patterns with prior evidence. Positive associations were observed for male gender, younger age, higher socioeconomic status, nonsmoking, and greater physical activity (moderate), whereas higher stress, depression, and chronic conditions were negatively associated with subjective health. Subsequently, subgroup analyses were conducted for the aging and super-aged areas using identical models.

### Comparison of Factors Between the 2 Groups

Using matched datasets for aging and super-aged areas, ordinal logistic regression was conducted with identical independent variables and ORs were derived to compare the factors influencing subjective health between the 2 groups (Table 3). Most variables showed significant associations consistent with the overall dataset, and the direction of these associations was largely similar across both regions. However, some differences emerged. Smoking status was not significantly associated with subjective health in the super-aged area (*P*=.06). Regarding nutrition label variables, label recognition was significant only in the aging area (*P*=.04), whereas label reading was significant only in the super-aged area (*P*=.05).

**Table 3.** Subgroup ordinal logistic regression analysis of factors associated with subjective health among older adults (≥65 y) in aging and super-aged areas of Korea.

Variable	Aging area		Super-aged area		*P* value^c^
	OR^a^ (95% CI)	*P* value^b^	Or (95% CI)	*P* value^b^	
Gender					
Male	1.29 (1.16‐1.42)	<.001	1.22 (1.05‐1.42)	.01	.56
Female	Reference	—^d^	Reference	—	—
Age (y)	0.99 (0.98‐0.99)	<.001	0.98 (0.97‐0.99)	<.001	.27
Education					
Under elementary	0.43 (0.36‐0.50)	<.001	0.45 (0.34‐0.58)	<.001	.81
Elementary school	0.51 (0.45‐0.57)	<.001	0.51 (0.44‐0.60)	<.001	.93
Middle school	0.60 (0.54‐0.67)	<.001	0.59 (0.50‐0.69)	<.001	.83
High school	0.76 (0.68‐0.84)	<.001	0.83 (0.72‐0.96)	.01	.30
College or over	Reference	—	Reference	—	—
Economic activity					
Yes	1.77 (1.65‐1.91)	<.001	1.63 (1.46‐1.82)	<.001	.22
No	Reference	—	Reference	—	—
Household income (1 million KRW)^e^	1.03 (1.01‐1.04)	<.001	1.03 (1.01‐1.05)	.006	.78
Smoking					
Yes	0.82 (0.74‐0.90)	<.001	0.86 (0.74‐1.01)	.06	.52
Nonsmoker	Reference	—	Reference	—	—
Drinking					
Almost never	0.62 (0.56‐0.69)	<.001	0.60 (0.50‐0.71)	<.001	.70
Less than once a month	0.87 (0.77‐0.99)	.03	0.76 (0.62‐0.95)	.01	.30
2‐4 times a month	0.90 (0.80‐1.02)	.11	0.91 (0.76‐1.09)	.31	.95
More than 2 times a week	Reference	—	Reference	—	—
Moderate physical activity (h)	1.02 (1.01‐1.03)	<.001	1.02 (1.01‐1.03)	<.001	.43
Walking (h)	1.04 (1.03‐1.05)	<.001	1.04 (1.02‐1.05)	<.001	.68
Subjective body shape					
Very thin	0.47 (0.39‐0.56)	<.001	0.41 (0.31‐0.55)	<.001	.49
Slightly thin	0.75 (0.69‐0.83)	<.001	0.77 (0.65‐0.91)	.002	.81
Average	Reference	—	Reference	—	—
Slightly obese	0.77 (0.72‐0.83)	<.001	0.84 (0.75‐0.96)	.008	.24
Very obese	0.40 (0.33‐0.47)	<.001	0.64 (0.48‐0.86)	.002	.005
Weight control experience					
Tried to lose weight	1.17 (1.07‐1.27)	<.001	1.04 (0.90‐1.20)	.60	.17
Tried to maintain	1.18 (1.09‐1.28)	<.001	1.25 (1.10‐1.42)	.001	.48
Tried to gain weight	0.92 (0.79‐1.07)	.26	1.17 (0.94‐1.44)	.16	.07^†f^
Never tried	Reference	—	Reference	—	—
Subjective stress					
Extremely stressed	0.18 (0.13‐0.24)	<.001	0.15 (0.10‐0.22)	<.001	.56
Much stressed	0.34 (0.31‐0.37)	<.001	0.34 (0.29‐0.40)	<.001	.97
Little stressed	0.63 (0.59‐0.67)	<.001	0.62 (0.56‐0.70)	<.001	.94
Stressless	Reference	—	Reference	—	—
Depression					
Yes	0.62 (0.55‐0.69)	<.001	0.56 (0.46‐0.67)	<.001	.39
No	Reference	—	Reference	—	—
Hypertension					
Yes	0.72 (0.68‐0.77)	<.001	0.65 (0.59‐0.72)	<.001	.09^†f^
No	Reference	—	Reference	—	—
Diabetes					
Yes	0.65 (0.61‐0.69)	<.001	0.58 (0.52‐0.65)	<.001	.10^†f^
No	Reference	—	Reference	—	—
Chewing difficulty					
Uncomfortable	0.68 (0.62‐0.74)	<.001	0.78 (0.68‐0.90)	.001	.11
Neutral	0.81 (0.74‐0.88)	<.001	0.99 (0.87‐1.13)	.89	.01
Comfortable	Reference	—	Reference	—	—
Subjective oral health					
Very good	8.50 (6.11‐11.82)	<.001	8.13 (5.47‐12.09)	<.001	.87
Good	3.82 (3.28‐4.45)	<.001	4.80 (3.67‐6.27)	<.001	.15
Fair	2.21 (1.93‐2.52)	<.001	2.76 (2.15‐3.55)	<.001	.12
Bad	1.73 (1.54‐1.96)	<.001	2.02 (1.59‐2.56)	<.001	.27
Very bad	Reference	—	Reference	—	—
Nutrition label recognition					
Yes	1.08 (1.00‐1.16)	.04	1.07 (0.96‐1.20)	.19	.93
No	Reference	—	Reference	—	—
Nutrition label reading					
Yes	1.09 (0.99‐1.19)	.09	1.16 (1.00‐1.35)	.046	.44
No	Reference	—	Reference	—	—
Unmet medical needs					
Yes (unmet)	0.55 (0.47‐0.64)	<.001	0.57 (0.46‐0.72)	<.001	.78
No (met)	Reference	—	Reference	—	—

a OR: odds ratio.

b *P* value within one region.

c *P* value between 2 region groups.

d Not applicable.

e 1 million KRW=US $700.

f † P < 0.10

### Comparison of ORs Between the 2 Groups

To assess regional differences in the impact of variables influencing subjective health, *z* tests comparing ORs between regions showed that perceiving oneself as “very obese” had a significantly weaker negative association with subjective health in the super-aged area (OR 0.64) than in the aging area (OR 0.40; *P*=.005). Hypertension (*P*=.09) and diabetes (*P*=.10) also showed marginally significant trends, with both conditions presenting lower ORs in the super-aged area.

## Discussion

### Key Findings

In this study, the proportion of respondents from super-aged areas who positively evaluated their subjective health (“very good” or “good”) was 28.9%. Furthermore, the proportion of positive subjective health evaluations in aging areas was 31.5%, higher than that in super-aged areas.

For drinking frequency, those consuming alcohol more frequently (“more than 2 times a week”) reported higher odds of positive health assessments compared to those drinking infrequently. Although the proportion of smokers was similar across regions, smoking status was not significantly associated with subjective health in super-aged areas.

Nutrition label recognition and reading exhibited distinct patterns across regions. In aging areas, nutrition label recognition was significantly associated with positive subjective health, whereas nutrition label reading was not. Conversely, in super-aged areas, despite higher recognition rates, recognition alone was not significantly related to subjective health, whereas nutrition label reading significantly influenced positive evaluations.

Hypertension and diabetes did not differ significantly between the 2 regions; both conditions tended to reduce the likelihood of positive subjective health evaluations more strongly in super-aged areas than in aging areas [24].

### Comparison With Previous Studies

The regional disparities observed in this study are consistent with prior research showing that rural regions tend to report lower subjective health than urban areas [25,26], likely due to the higher proportion of rural communities within super-aged areas [27].

Demographic characteristics, health behaviors, health status, and unmet health care needs were significantly associated with subjective health, consistent with previous studies [11,15,28-30]. Similar patterns were also observed for chronic conditions such as hypertension and diabetes, consistent with prior findings [31].

Although research investigating the association between nutrition labels and subjective health is limited, Dumotier et al [32] reported that recognizing and using nutrition labels significantly influenced dietary habits and obesity. Similarly, Jang’s study [33] indicated that health literacy significantly improved subjective health status.

### Plausible Underlying Mechanisms

Although previous studies generally suggest that higher drinking frequency is negatively associated with health [14], some studies have found that moderate drinking frequency is positively linked to subjective health [34], possibly due to the social interactions accompanying drinking [35]. The lack of association between smoking and subjective health may indicate that factors beyond individual smoking behavior, such as government tobacco control policies or the socioeconomic characteristics of smokers in different regions, could have influenced self-reported health, potentially contributing to regional differences [36,37].

In Korea, approximately 84% of older adults have at least one chronic condition, many of which require dietary modifications, making it particularly important for this population to utilize information on food labels [38]. However, a high proportion of older adults report difficulties in reading and interpreting food labels [39]. Such challenges may stem from age-related declines in numeracy and attention, which can reduce the ability to process and apply nutritional information effectively [40].

Nutrition knowledge plays a key role as a predictor of both comprehension and utilization of nutrition labels [41]. In Korea, nutrition education for older adults is mainly provided through public health centers and senior welfare centers, yet access to such programs remains limited in super-aged areas. These areas are disproportionately rural, where older residents often face longer travel distances to health care facilities and have fewer community-based resources, leading to reduced participation in preventive programs and health education initiatives.

### Limitations

This study has several limitations. First, although the KCHS is a nationally representative dataset, its cross-sectional design allows for the assessment of associations but not causality between subjective health and its related factors. Longitudinal studies are needed to establish causal relationships and to address potential reverse causation. Second, regional classification based solely on aging rates may not fully reflect the complex social and environmental characteristics of each community. However, by applying this classification at the district level rather than using broad administrative boundaries, the study was able to capture substantial variations in aging rates and subjective health. This finer granularity likely improved the sensitivity of the analysis and better reflected Korea’s ongoing demographic transitions.

### Policy Implications

Improving subjective health is essential for increasing healthy life expectancy and enhancing quality of life in South Korea, a nation experiencing rapid population aging. It has also been designated as a specific indicator within Korea’s 5th National Health Plan (HP2030), highlighting it as a national priority requiring focused intervention [42]. The lower proportion of older adults reporting positive subjective health in super-aged areas, compared with both the 2023 KCHS national average (47.6%) and the HP2030 target (34.7%), underscores the need for targeted public health interventions.

Given that 77% of households with older adults in Korea report that older adults are the primary purchasers of food [43], the ability to accurately read and interpret nutrition labels is particularly important for this population. In Korea, front-of-package (FOP) nutrition labeling has not yet been implemented, whereas countries such as Canada have adopted standardized FOP symbols to help consumers quickly identify foods high in sodium, sugar, or saturated fat [44]. Such government-endorsed labeling has been shown to improve the accuracy and speed of consumers’ food choices and is particularly beneficial for populations with lower health literacy, including older adults [45]. Introducing a similar labeling policy in Korea could therefore promote informed food selection and facilitate the effective use of nutrition labels in daily dietary decisions.

Complementary to such policy measures, nutrition education remains essential, particularly in super-aged areas where accessibility to educational programs remains limited. Previous research has shown that when older adults focus on specific nutrients, their accuracy in interpreting nutrition labels can be comparable to that of younger adults [40]. This suggests that targeted and nutrient-specific education can effectively enhance comprehension among older adults. Notably, in this study, hypertension and diabetes, although similarly prevalent across regions, showed a stronger negative association with subjective health in super-aged areas than in aging areas [24], indicating that nutrition label education emphasizing disease-related nutrients may be particularly beneficial in super-aged areas, as supported by previous evidence that tailored nutrition education significantly improved dietary knowledge and behaviors among the elderly [46].

When combined with FOP labeling and community-based education through local health centers, such tailored nutrition programs could strengthen dietary awareness, promote active label use, and help reduce health disparities among older adults [47].

### Conclusions

This study revealed regional differences in factors influencing subjective health among older adults in Korea. In super-aged areas, reading and understanding nutrition labels, rather than merely recognizing them, were more strongly associated with positive subjective health, while the negative effects of hypertension and diabetes were also more pronounced. These findings highlight the need for region-specific public health strategies that include tailored nutrition education focusing on disease-related nutrients to improve subjective health among older adults.

## Supplementary material

10.2196/80189 Multimedia Appendix 1 Demographic characteristics of older adults (≥65 years) in aging and super-aged areas of Korea before propensity-score matching.

10.2196/80189 Multimedia Appendix 2 Ordinal logistic regression analysis of factors associated with subjective health among older adults (≥65 y) in Korea.

10.2196/80189 Checklist 1 STROBE checklist.

## Abbreviations

FOP: front-of-package
KCHS: Korea Community Health Survey
KDCA: Korea Disease Control and Prevention Agency
OECD: Organization for Economic Co-operation and Development
OR: odds ratio
PSU: primary sampling unit

## References

[1] Population projections: estimated population by major age groups (working-age population, older population, etc.), national data [Web page in Korean]. Korean Statistical Information Service (KOSIS).

[2] Koohsari MJ, Nakaya T, Oka K (2018). Activity-friendly built environments in a super-aged society, Japan: current challenges and toward a research agenda. Int J Environ Res Public Health.

[3] Jones RS (2024). Addressing demographic headwinds in Japan: a long-term perspective. https://www.oecd.org/content/dam/oecd/en/publications/reports/2024/04/addressing-demographic-headwinds-in-japan-a-long-term-perspective_85b9a67f/96648955-en.pdf.

[4] Robine JM, Saito Y, Jagger C (2009). The relationship between longevity and healthy life expectancy. Qual Ageing Older Adults.

[5] (2023). World health statistics 2023: monitoring health for the SDGs, sustainable development goals. https://www.who.int/publications/i/item/9789240074323.

[6] (2023). Healthy life expectancy statistics: healthy life expectancy at a glance, 2022 [Report in Korean]. https://www.khepi.or.kr/board/view?linkId=1007632&menuId=MENU01592&no1=2&pageNum=1&rowCnt=10.

[7] Browning CJ, Leith KH, Thomas SA (2023). Editorial: economic and social factors affecting the health of older adults. Front Public Health.

[8] (2016). Is subjective health reliable as a proxy variable for true health? A comparison of self-rated health and self-assessed change in health among middle-aged and older South Koreans [Article in Korean]. J Health Soc Sci.

[9] (2024). OECD health statistics 2024 (summary) booklet [Report in Korean]. https://www.mohw.go.kr/board.es?mid=a10411010100&bid=0019&act=view&list_no=1483195.

[10] Wang J, Zhang L, Wang S, Zhang L (2023). Living arrangements, health lifestyles, and health outcomes among Chinese oldest-old. Front Public Health.

[11] Kwon MJ (2021). Factors influencing convergence quality of life of the elderly according to economic activity [Article in Korean]. J Korea Converg Soc.

[12] Barsha RAA, Najand B, Zare H, Assari S (2024). Immigration, educational attainment, and subjective health in the United States. J Ment Health Clin Psychol.

[13] Lin CC, Kuo CT, Tsai MR (2022). Association of functional, interactive, and critical health literacy with good self-rated health among Taiwanese community-dwelling older adults. Geriatr Nurs.

[14] Lee JC, Park JS, Kim GH (2011). The effects of stress, quality of life and family relationship of smokers and drinkers on tobacco and alcohol use: focusing on the mediating effects of self-rated health [Article in Korean]. Korean Public Health Res.

[15] Cho JO, Park SH (2022). Relationship between exercise practice and subjective health status in the elderly. Soc Occup Ther Aged Dement.

[16] Chun HJ, Kim HY, Chun KK (2019). The effects of objective characteristics and subjective evaluation of community on depression of adults - the case of Seoul city. J Community Welf.

[17] Lee MS (2005). Health inequalities among Korean adults: socioeconomic status and residential area differences [Article in Korean]. Korean J Sociol.

[18] Liao Y, Cheng X, Li Z, Li Y (2023). The mediating role of physical activity and health status between a health-supportive environment and well-being: a cross-sectional study. Front Public Health.

[19] Yen IH, Michael YL, Perdue L (2009). Neighborhood environment in studies of health of older adults: a systematic review. Am J Prev Med.

[20] (2025). Age-friendly baseline assessment of Taebaek city. https://extranet.who.int/agefriendlyworld/wp-content/uploads/2025/09/Age-freindly-Baseline-Assessment-of-Taebaek-City-1.pdf.

[21] Jang EJ, Ahn J, Jung SY (2013). Methods for the Control of Measured Confounders in Outcomes Research.

[22] Enforcement Rule of the Bioethics and Safety Act (Ordinance of the Ministry of Health and Welfare no1115) [Web page in Korean]. Ministry of Health and Welfare, Republic of Korea.

[23] Lee SW, Acharya KP (2022). Propensity score matching for causal inference and reducing the confounding effects: statistical standard and guideline of Life Cycle Committee. Life Cycle.

[24] Mu R (2014). Regional disparities in self-reported health: evidence from Chinese older adults. Health Econ.

[25] Saha A, Rahaman M, Mandal B, Biswas S, Govil D (2022). Rural urban differences in self-rated health among older adults: examining the role of marital status and living arrangements. BMC Public Health.

[26] Zhang YL, Wu BJ, Chen P, Guo Y (2021). The self-rated health status and key influencing factors in middle-aged and elderly: evidence from the CHARLS. Medicine (Baltimore).

[27] Kang SH (2015). Regional differences in self-perceived health [Article in Korean]. Health Soc Sci.

[28] Choi IH (2022). Factors affecting the self-rated health status in rural residents-using lalonde health field model [Article in Korean]. J Healthc Life Sci.

[29] Lee JW (2020). Effect of unmet healthcare needs on quality of life [Article in Korean]. J Korea Acad Ind Coop Soc.

[30] Venegas-Sanabria LC, Moreno-Echeverry MM, Borda MG, Chavarro-Carvajal DA, Cano-Gutierrez CA (2023). Oral health and self-rated health in community-dwelling older adults in Colombia. BMC Oral Health.

[31] Sahril N, Chan YM, Chan YY (2023). Poor self-rated health and associated factors among older persons in Malaysia: a population-based study. Int J Environ Res Public Health.

[32] Dumoitier A, Abbo V, Neuhofer ZT, McFadden BR (2019). A review of nutrition labeling and food choice in the United States. Obes Sci Pract.

[33] Jang YE (2023). Influence of the health literacy on subjective health status [Article in Korean]. J Korea Acad Ind Cooper Soc.

[34] Nam YH, Nam JR (2011). A study of the factors affecting the subjective health status of elderly people in Korea [Article in Korean]. Korean J Fam Walf.

[35] Lim JS, Kim D, Yoo J, Jung H, Park JH (2024). A longitudinal study of the impact of social activity on drinking in older adults [Article in Korean]. J Ind Converg.

[36] Park S, Ahn J, Lee BK (2015). Self-rated subjective health status Is strongly associated with sociodemographic factors, lifestyle, nutrient intakes, and biochemical indices, but not smoking status: KNHANES 2007-2012. J Korean Med Sci.

[37] Verropoulou G (2009). Key elements composing self-rated health in older adults: a comparative study of 11 European countries. Eur J Ageing.

[38] Post RE, Mainous AG, Diaz VA, Matheson EM, Everett CJ (2010). Use of the nutrition facts label in chronic disease management: results from the National Health and Nutrition Examination Survey. J Am Diet Assoc.

[39] Deakin TA (2011). Consumers find food labels confusing and too small to read. Pract Diab Int.

[40] Miller LMS, Applegate E, Beckett LA, Wilson MD, Gibson TN (2017). Age differences in the use of serving size information on food labels: numeracy or attention?. Public Health Nutr.

[41] Miller LMS, Cassady DL (2015). The effects of nutrition knowledge on food label use. a review of the literature. Appetite.

[42] (2021). 5th comprehensive national health promotion plan (health plan 2030, 2021~2030) [Report in Korean]. https://www.khepi.or.kr/board/view?boardStyle=Gallery&categoryId=&contents1=&continent=&country=&linkId=1002769&menuId=MENU01320&no1=27&pageNum=1&rowCnt=8.

[43] (2024). Food consumption behavior survey 2023 [Report in Korean]. https://repository.krei.re.kr/bitstream/2018.oak/31053/1/E16-2023.pdf.

[44] (2022). Regulations amending the food and drug regulations (nutrition symbols, other labelling provisions, vitamin D, and hydrogenated fats or oils), SOR/2022-168. Government of Canada.

[45] (2018). Consumer research on front-of-package nutrition labeling. https://publications.gc.ca/collections/collection_2018/sc-hc/H164-252-2018-eng.pdf.

[46] Wallace R, Lo J, Devine A (2016). Tailored nutrition education in the elderly can lead to sustained dietary behaviour change. J Nutr Health Aging.

[47] Fan L, Wang Z, Zhao Y, Ma Y (2023). Urban-rural disparities in knowledge, use and perceived benefits of nutrition labels in China: evidence from 10 provinces. Nutrients.

