# Supplementary material for: Regional Disparities in Factors Associated With Subjective Health Among Older Adults in Aging and Super-Aged Areas of Korea: Nationwide Cross-Sectional Study
Source: JMIR Public Health Surveill. 2026 Mar 2;12:e80189. doi: 10.2196/80189 (PMC13384912; doi:10.2196/80189)
Supplement: Multimedia Appendix 1 [file publichealth-v12-e80189-s001.docx]

Supplement 1. Demographic characteristics of older adults (≥65 years) in aging and super-aged areas of Korea before propensity-score matching

| **Variable** | **Full data** | | |
| --- | --- | --- | --- |
|  | **Aging Area**^a^  **(N^a^ = 19,759)** | **Super-aged Area**  **(N = 159,782)** | **p-value^b^(SMD)^c^** |
| **Gender, n (%)** |  |  | .24(.005) |
| Male | 2129570 (45.7) | 4437643 (45.3) |  |
| Female | 2530817 (54.3) | 5363718 (54.7) |  |
| Age (years), mean (SD) | 73.3 (6.6) | 74.3 (6.8) | <.001(-.18) |
| **Age (years), n (%)** |  |  | <.001 |
| 65-74 | 2874637 (61.7) | 5416794 (55.3) |  |
| Over 75 | 1785750 (38.3) | 4384567 (44.7) |  |
| **Household type, n (%)** |  |  | <.001(.26) |
| Living alone | 907533 (19.5) | 2377931 (24.3) |  |
| With a spouse or other | 2249312 (48.3) | 5336151 (54.4) |  |
| Two Generations | 1060453 (22.8) | 1626325 (16.6) |  |
| Three Generations | 443089 (9.5) | 460953 (4.7) |  |
| **Marital Status, n (%)** |  |  | <.001 |
| Yes, Cohabitation | 3015938 (64.7) | 6364428 (64.9) |  |
| Separation or Divorced | 369845 (7.9) | 611694 (6.2) |  |
| Bereavement | 1240621 (26.6) | 2758372 (28.1) |  |
| No | 33983 (0.7) | 66866 (0.7) |  |
| **Education, n (%)** |  |  | <.001(.39) |
| Under Elementary | 355139 (7.6) | 1267084 (12.9) |  |
| Elementary | 1341033 (28.8) | 3862032 (39.4) |  |
| Middle school | 956423 (20.5) | 1916030 (19.5) |  |
| High school | 1243294 (26.7) | 1896950 (19.4) |  |
| College or over | 764498 (16.4) | 859265 (8.8) |  |
| Economic Activity, yes, n (%) | 1248079 (26.8) | 3804498 (38.8) | <.001 |
| Household income, 1 million KRW^d^, mean (SD) | 264.0 (248.5) | 191.0 (187.9) | <.001(.30) |

^a^Aging areas were defined as regions with aging rates ≤14%, and super-aged areas as those with ≥20%

^b^Rao-Scott Chi-square tests for Categorical Variable, Independent sample t-tests for Continuous Variable.

^c^ Standardized mean differences(SMD) for variables used in propensity score matching

^d^1 million KRW=US $700.
