# Supplementary material for: Regional Disparities in Factors Associated With Subjective Health Among Older Adults in Aging and Super-Aged Areas of Korea: Nationwide Cross-Sectional Study
Source: JMIR Public Health Surveill. 2026 Mar 2;12:e80189. doi: 10.2196/80189 (PMC13384912; doi:10.2196/80189)
Supplement: Multimedia Appendix 2 [file publichealth-v12-e80189-s002.docx]

Supplement 2. Ordinal logistic regression analysis of factors associated with subjective health among older adults (≥65 years) in Korea

| **Variable** | | **Full data^a^** |  |
| --- | --- | --- | --- |
|  |  | **OR^b^ (95% CI^c^)** | **p-value** |
| **Gender** | |  |  |
|  | Male | 1.27 (1.15-1.40) | <.001 |
|  | Female | 1.00 (reference) |  |
| **Age (years)** | | 0.99 (0.98-0.99) | <.001 |
| **Education** | |  |  |
|  | Under Elementary | 0.43 (0.37-0.51) | <.001 |
|  | Elementary school | 0.51 (0.46-0.56) | <.001 |
|  | Middle school | 0.60 (0.54-0.66) | <.001 |
|  | High school | 0.79 (0.72-0.86) | <.001 |
|  | College or over | 1.00 (reference) | - |
| **Economic Activity** | |  |  |
|  | Yes | 1.68 (1.56-1.80) | <.001 |
|  | No | 1.00 (reference) | - |
| **Household Income (1 million KRW)** | | 1.03 (1.02-1.04) | <.001 |
| **Smoking** | |  |  |
|  | Yes | 0.84 (0.76-0.92) | <.001 |
|  | Non-Smoker | 1.00 (reference) | - |
| **Drinking** | |  |  |
|  | Almost never | 0.62 (0.55-0.68) | <.001 |
|  | Less than Once a month | 0.84 (0.74-0.95) | .006 |
|  | 2-4 times a month | 0.91 (0.81-1.02) | .12 |
|  | Over 2 times a week | 1.00 (reference) | - |
| **Moderate Physical Activity (hour)** | | 1.02 (1.01-1.03) | <.001 |
| **Walking (hour)** | | 1.04 (1.03-1.05) | <.001 |
| **Subjective Body Shape** | |  |  |
|  | Very Thin | 0.44 (0.37-0.53) | <.001 |
|  | Slightly Thin | 0.76 (0.69-0.84) | <.001 |
|  | Average | 1.00 (reference) | - |
|  | Slightly Obese | 0.81 (0.75-0.87) | <.001 |
|  | Very Obese | 0.48 (0.40-0.57) | <.001 |
| **Weight Control Experience** | |  |  |
|  | Tried to lose weight | 1.13 (1.04-1.23) | .005 |
|  | Tried to maintain | 1.22 (1.12-1.32) | <.001 |
|  | Tried to gain weight | 1.02 (0.89-1.18) | .76 |
|  | Never tried | 1.00 (reference) | - |
| **Subjective Stress** | |  |  |
|  | Extremely Stressed | 0.17 (0.13-0.21) | <.001 |
|  | Much Stressed | 0.34 (0.31-0.37) | <.001 |
|  | Little Stressed | 0.62 (0.58-0.67) | <.001 |
|  | Stressless | 1.00 (reference) | - |
| **Depression** | |  |  |
|  | Yes | 0.60 (0.54-0.67) | <.001 |
|  | No | 1.00 (reference) | - |
| **Hypertension** | |  |  |
|  | Yes | 0.69 (0.65-0.74) | <.001 |
|  | No | 1.00 (reference) | - |
| **Diabetes** | |  |  |
|  | Yes | 0.62 (0.58-0.66) | <.001 |
|  | No | 1.00 (reference) | - |
| **Chewing Difficult** | |  |  |
|  | Uncomfortable | 0.73 (0.66-0.79) | <.001 |
|  | Neutral | 0.88 (0.81-0.96) | .003 |
|  | Comfortable | 1.00 (reference) | - |
| **Subjective Oral Health** | |  |  |
|  | Very Good | 8.21 (6.24-10.79) | <.001 |
|  | Good | 4.19 (3.59-4.89) | <.001 |
|  | Fair | 2.42 (2.10-2.78) | <.001 |
|  | Bad | 1.83 (1.61-2.08) | <.001 |
|  | Very Bad | 1.00 (reference) | - |
| **Nutrition Label Recognition** | |  |  |
|  | Yes | 1.07 (1.00-1.14) | .06 |
|  | No | 1.00 (reference) | - |
| **Nutrition Label Reading** | |  |  |
|  | Yes | 1.13 (1.03-1.23) | .009 |
|  | No | 1.00 (reference) | - |
| **Unmet Medical Needs** | |  |  |
|  | Yes (Unmet) | 0.56 (0.48-0.64) | <.001 |
|  | No (Met) | 1.00 (reference) | - |

^a^ Full data includes Aging area(n=19,759) and Super-aged area(n=18,574)

^b^ OR = Odds Ratio

^c^ 95% Confidence Interval
